# Technical considerations for cost-effective transposon directed insertion-site sequencing (TraDIS)

**DOI:** 10.1038/s41598-024-57537-6

**Published:** 2024-03-21

**Authors:** Yasuhiro Kyono, Madeline Tolwinski, Stephanie A. Flowers

**Affiliations:** Department of Pharmacy Practice, College of Pharmacy, University of Illinois at Chicago, 833 S Wood St. #125B, Chicago, IL 60612 USA

**Keywords:** Transposon insertion sequencing, *Escherichia coli*, Electroporation, Illumina library preparation, Genetics, Microbiology, Molecular biology

## Abstract

Transposon directed insertion-site sequencing (TraDIS), a variant of transposon insertion sequencing commonly known as Tn-Seq, is a high-throughput assay that defines essential bacterial genes across diverse growth conditions. However, the variability between laboratory environments often requires laborious, time-consuming modifications to its protocol. In this technical study, we aimed to refine the protocol by identifying key parameters that can impact the complexity of mutant libraries. Firstly, we discovered that adjusting electroporation parameters including transposome concentration, transposome assembly conditions, and cell densities can significantly improve the recovery of viable mutants for different *Escherichia coli* strains. Secondly, we found that post-electroporation conditions, such as recovery time and the use of different mediums for selecting mutants may also impact the complexity of viable mutants in the library. Finally, we developed a simplified sequencing library preparation workflow based on a Nextera-TruSeq hybrid design where ~ 80% of sequenced reads correspond to transposon-DNA junctions. The technical improvements presented in our study aim to streamline TraDIS protocols, making this powerful technique more accessible for a wider scientific audience.

## Introduction

Transposon directed insertion-site sequencing (TraDIS) is a powerful high-throughput assay used to identify essential bacterial genes in various growth conditions^1^. This technique operates by randomly integrating Tn5 mini-transposons into the bacterial genome through the action of the transposase enzyme, a process that disrupts or alters gene function. While widely applied, TraDIS protocols are not standardized and often require custom modifications to suit the unique circumstances of different laboratories, such as variations in available equipment and bacterial strains. These adjustments can be particularly time-consuming and labor-intensive for those new to the technique.

Our current study aims to streamline the construction of complex mutant libraries by identifying key parameters that enhance labor efficiency and practicality. We employed *Escherichia coli* strains from diverse sources, including freshwater environments, the human gut, and laboratory settings, to demonstrate the versatility and applicability of our methods. Our investigation included optimizing electroporation conditions to improve transposon insertion and mutant recovery. Specifically, we fine-tuned the concentration of the transposome, evaluated the effect of varying transposon DNA quantities during transposome assembly, and explored how cell density affects the outcomes of electroporation reactions.

Following the optimization of electroporation conditions, our study investigated the impact of post-electroporation parameters on the diversity of mutants with unique insertion sites. We assessed the length of recovery time after electroporation and compared the efficacy of different mediums for selecting mutants, namely agar plates versus liquid broth. While using liquid broth is a simpler approach, it potentially compromises the complexity of the recovered mutant library, as mutants compete for shared resources before the culture can be collected. These comparisons aimed to determine the most effective method for constructing complex mutant libraries while considering factors such as labor efficiency and practicality.

Another important aspect of TraDIS assay is a robust PCR protocol to enrich transposon-DNA junctions, while minimizing amplification of non-specific fragments without such junctions. Prior strategies include the use of splinkerette adaptors followed by a sequencing run with custom primers and operation protocols^2,3^. However, this strategy requires a dedicated run for TraDIS libraries, which can limit the use of a higher capacity, more cost-effective sequencer like NovaSeq. Another strategy is a nested PCR approach using ‘all-in-one’ primers that combine both the Illumina flow cell and transposon-specific sequences^4,5^. Although this method is effective, it involves the use of long primers (exceeding 60 base pairs), which often necessitates more expensive synthesis. Also, if one were to use a different transposon for the assay, this requires the synthesis of a new set of ‘all-in-one’ primers. To this end, we present a PCR strategy that simplifies the library preparation workflow. Our library construction scheme is compatible with standard TruSeq/Nextera indexing primers used for other Illumina assays, thereby reducing the overall cost.

## Methods

### Bacterial strains (Supplemental Table 1)

We utilized three strains of *Escherichia coli*, each selected for its unique origin and characteristics. AB_00116 was isolated from a freshwater environment and provided by Aaron Best's laboratory at Hope College, Holland, Michigan, USA. MS 69-1 was obtained from BEI Resources (HM347) and originally isolated from the colon of a control patient with abnormal histology in New York, New York, USA. D43HC1 was derived from a laboratory *E. coli* strain ATCC25922, previously characterized as multi-drug resistant after being exposed to a gut relevant-concentration of the antipsychotic drug quetiapine in vitro for 6 weeks^6^. These strains were chosen to represent a diverse range of environmental and physiological conditions, thereby ensuring the robustness and applicability of our experimental findings across different bacterial backgrounds.

### Synthesis of transposon encoding the kanamycin resistance gene (KAN2)

Using the Twist Bioscience Gene Fragments service, we ordered a custom DNA fragment corresponding to the KAN2 transposon (1221 bp), whose full sequence is available on the product manual for the EZ-Tn5 < KAN-2 > Tnp Transposome Kit (Lucigen #TSM99K2). We PCR amplified 1 ng of the fragment with Q5® High-Fidelity 2X Master Mix (NEB #M0492) using a PCR primer set: Fwd 5′—/5′Phos/CTGTCTCTTATACACATCTCAACCATCATCGA—3′ and Rev 5′—/5′Phos/CTGTCTCTTATACACATCTCAACCCTGAAGCT—3′ and the following PCR cycle parameters: 98 °C for 30 s, 18 cycles of (98 °C for 10 s, 69 °C for 30 s, 72 °C for 45 s), and 72 °C for 2 min (50 uL reactions in triplicate). After confirming the amplicon size by gel electrophoresis, we purified the amplicon using the DNA Clean & Concentrator-5 kit (Zymo Research #D4013). To increase the DNA concentration in the final eluate, we used three purification columns (i.e. one column for each PCR reaction). At the elution step, we eluted the first column with 25 uL of the fresh elution buffer for the DNA Clean & Concentrator-5 kit (10 mM Tris-HCol, ph 8.5, 0.1 mM EDTA). For the second and third columns, we used the first and second eluates as the elution medium.

### Transposome assembly

We purchased the unloaded Tagmentase (Tn5 transposase) from Diagenode (#C01070010-10; 2 mg/mL = 37.52 uM), and adjusted the protein concentration to 1 uM by diluting it in the storage buffer described in the manual for the EZ-Tn5 Transposase (LGC Biosearch Technologies) (50 mM Tris–HCl (pH 7.5), 100 mM NaCl, 0.1 mM EDTA, 0.1% Triton^®^ X-100 (Rohm & Haas) and 1 mM DTT). To assemble the transposome, we mixed 1 part of the KAN2 transposon DNA solution, 2 parts of the diluted Tn5 at 1 uM, and 1 part of 100% glycerol, and incubated in a thermocycler at 23 °C for 60 min before use (or stored at − 20 °C if not used immediately).

### Electroporation experimental setup

#### Basic workflow

To make *E. coli* electrocompetent, we first inoculated a colony of bacteria in 3 mL of LB medium and shake-incubated at 37 °C overnight. Next day, we transferred the overnight culture in fresh LB medium (1/100 dilution) and shake-incubated again at 37 °C until the optical density at 600 nm (OD_600_) of the culture reached ~ 0.4. Cells were harvested by centrifugation at 4000*g* for 10 min at 4 °C, washed twice in 2/3 culture volume of sterile water (4 °C) (centrifugation after each wash), and then resuspended in 1/100 culture volume of sterile water (4 °C) (e.g. for 45 mL of culture, 30 mL water was used for each wash, and 450 uL water was used for final resuspension). Cells and electroporation cuvettes (1 mm gap) were kept on ice until electroporation. We used BioRad Gene Pulser to electroporate the bacteria using the following parameters: 2000 V, 25 uF and 200 Ω. Immediately after electroporation, we added 1 mL of SOC medium, pipette up and down several times, and transferred to a sterile 2 mL screw-cap tube for recovery in a shaker incubator at 37 °C (with tubes placed in a horizontal position for better aeration). We recorded the time constant for each electroporation reaction.

### Analyzing the effect of selection medium (agar plate vs. liquid culture) on mutant library complexity

After 1 h of recovery from electroporation, we selected mutants as follows: *Agar plates*: We spread one portion of the culture on LB agar plates containing 40 µg/mL kanamycin (each plate received an appropriate volume of culture to generate 1000–2000 CFUs), and incubated at 37 °C overnight. On the next day, we first measured CFUs using a ProtoCOL3 automatic colony counter (Synbiosis), and then scraping off colonies from agar plates using a sterile cell spreader and 1 mL of LB. We recovered an equal number of mutants for each strain under investigation. We extracted DNA from a dilute sample of the pooled culture using the DNeasy Blood & Tissue kit (Qiagen) according to the manufacturer's instructions. *Liquid culture*: We transferred the equal volume of the culture directly into an Erlenmyer flask with fresh LB medium containing 40 µg/mL kanamycin (the volume of LB-kanamycin medium was 100X of the transferred culture volume). We shake-incubated the flask (175 r.p.m.) at 37 °C, and collected 1 mL sample when the OD_600_ of the culture reached 0.4, 0.8, and 1.2, which corresponds to early-, mid-, and late-log growth phase, respectively. We extracted DNA from each sample using the DNeasy Blood & Tissue kit (Qiagen) according to the manufacturer's instructions.

### Analyzing the effect of recovery time on mutant library complexity

After electroporation and addition of SOC medium for each strain, we combined multiple reactions into one to normalize the potential difference in electroporation efficiency of each reaction, and then immediately split them into equal portions among 2 mL screw-cap tubes. All portions were shake-incubated in parallel at 37 °C, and they were spread onto LB-kanamycin plates at designated time points. All plates were incubated at 37 °C overnight. Each experiment was performed in duplicate. On the next day, we first measured CFUs using the ProtoCOL3 automatic colony counter, and then scraped off colonies from agar plates using a sterile cell spreader and 1 mL of LB. We extracted DNA from a dilute solution of the pooled culture using the DNeasy Blood & Tissue kit (Qiagen) according to the manufacturer’s instructions.

### Statistics

Statistical analysis involved applying a two-way analysis of variance (ANOVA) to assess significance, followed by Tukey’s Honest Significant Difference test for post hoc comparisons.

### Construction of DNA libraries and sequencing

We sonicated extracted DNA samples using the Covaris S2 sonicator (parameters: Intensity – 5; Duty Factor – 10; time – 90 s) to fragment the genome into 300–400 bp pieces. Proper fragmentation was verified via gel electrophoresis. We used the NEBNext Ultra II DNA Library Prep Kit for Illumina (NEB #E7103) to constructed DNA library (input = 500 ng) according to the manufacturer's protocol (version 7.0_9/22) with the following adjustments: (1) During fragment size selection, we used 22.5 µL (Step 3A.2) and 10 µL (Step 3A.6) of the NEBNext Sample Purification Beads; (2) At “Step 4.1 PCR Amplification” we enriched the transposon-DNA junction using the following PCR parameters: 1 cycle of 98℃ for 30 s, 22 cycles of [98 °C for 10 s, 65 °C for 75 s], and 1 cycle of 65 °C for 5 min (primer sequences are listed in Supplemental Table 2; and (3) We eluted the resulting PCR products using 52.5 uL of 10 mM Tris (pH 8.0) (Step 5.9) and collected 50 uL of the eluate in Step 5.11. We measured the concentration of each eluate using Qubit reagent (Invitrogen #Q32851) and adjusted concentrations of each sample to 4 ng/uL with 10 mM Tris (pH 8.0). Using 20 ng of 1st PCR product as input material, we conducted index PCR (50 uL reaction) with 2X KAPA HiFi HotStart ReadyMix (Roche Diagnostics #07958927001), Illumina Nextera/TruSeq P5 and TruSeq P7 index primers, and the following PCR parameters: 1 cycle of 95 °C for 3 min, 8 cycles of [95 °C for 10 s, 55 °C for 30 s, 72 °C for 30 s], and 1 cycle of 72 °C for 5 min. We purified the resulting product with the HighPrep PCR beads (MagBio Genomics #AC-60005) according to the manufacturer's protocol (version v2.0) using the beads-to-sample ratio of 1:1.12. We pooled libraries in equimolar concentration and sequenced on the Illumina NovaSeq 6000 platform (150 bp PE).

### Sequencing data analysis

We first subjected fastq reads to two rounds of filtering using the *cutadapt* software (ver. 4.1) to remove reads that did not have expected fragment layout. For the first round of filtering, we trimmed a total of 29 bp sequences from Read 1 (5′—GCATGCAAGCTTCAGGGTTGAGATGTGTA—3′), which corresponded to the PCR anchor (20 bp) and the 5′ portion of the 19-bp Tn5 mosaic end (ME) sequence (9 bp), with a maximum of 10% mismatches allowed. For the second round, we trimmed the remaining 10-bp ME sequence (5′—TAAGAGACAG—3′), with no mismatch allowed. In both rounds of filtering, we discarded reads that did not pass the filtering criteria (‘–discard-untrimmed’), and corresponding read mates were discarded from Read 2 fastq files. We mapped the filtered reads (Read 1 only) to respective reference FASTA in both forward and reverse complement orientations using the *bwa* software (version 0.7.17-r1188) with default options.

We defined the transposon-DNA junctions, which is equivalent to the number of unique mutants in the library, as the leftmost coordinate of the mapped reads, and we used a custom bash script to determine this. We first sorted the mapped reads based on their genomic coordinates using the *samtools* software (version 1.14) and filtered for reads that align to forward orientation (FLAG = 0 in the second column). We then de-duplicated reads with identical coordinates (chromosome and the leftmost position of the mapped reads) and counted the number of unique reads as ‘putative junctions’. We filtered out putative junctions with low number of reads (< 1 count per million reads mapped), and then classified the remaining junctions as ‘true junctions’, which are reported in the Results section. The code used for this analysis can be found here: https://github.com/StephanieAFlowers/TraDIS_techical_paper.git.

## Results

### Diluting transposome improves electroporation efficiency

**Figure 1:**
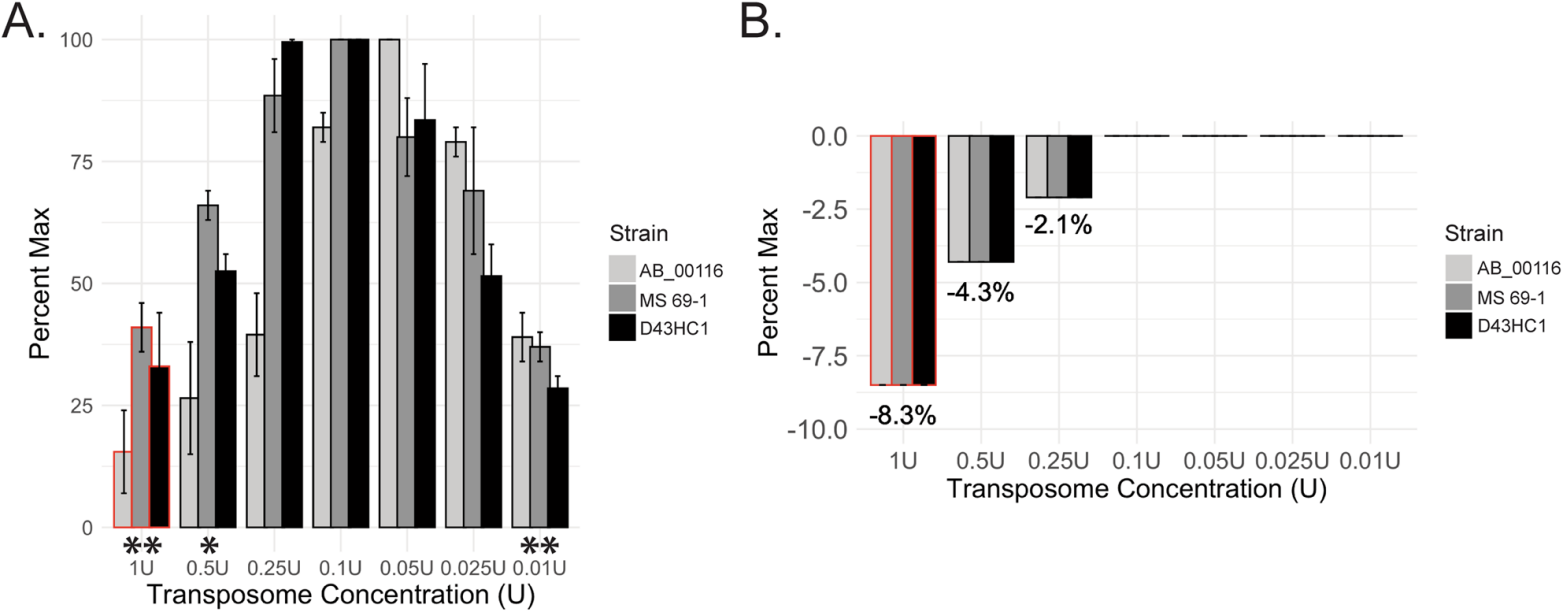
Diluting transposome improved electroporation efficiency. We electroporated our *E. coli* cohort with various amounts of Tn5 transposome and measured CFUs the following day. (**A**) Bars indicate normalized CFUs as a function of transposome concentration (1U = 0.005 µM). Error bars indicate variance among replicates (p = 3.95e–08). (**B**) Bars indicate % change from the theoretically maximum time constant (τ) during electroporation as a function of transposome concentration (1U = 0.005 µM). In both panels, bars with red highlights indicate the recommended transposome concentration in a standard TraDIS protocol (EZ-Tn5 Transposase manual). *p < 0.01, **p < 0.001.

We examined how electroporation efficiency is affected by the amount of transposome in electroporation reaction (Fig. 1A). To analyze this, we serially diluted the most concentrated transposome (1 U) to 0.5, 0.25, 0.1, 0.05, 0.025, and 0.01 U, mixed into electrocompetent cells, and assessed the electroporation efficiency by measuring CFUs the next day. All conditions for each strain were performed in duplicate. We found that CFUs increased as we used more diluted solutions of transposome, peaked around 0.05 U for AB_00116 strain or 0.1 U for MS 69–1 strain, and then declined. The effect of transposome concentration on electroporation efficiency, assessed via two-way ANOVA, was significant (p = 3.95e–08). The time constant (τ) is measured by the product of resistance and capacitance and often correlates with electroporation efficiency. We found that the time constants for reactions containing 1, 0.5, and 0.25 U transposome were 8.7%, 4.3%, and 2.2% lower compared to those containing less than 0.25 U, which correlated with lower CFUs (Fig. 1B).

### Higher quantity of transposon DNA during transposome assembly improves electroporation efficiency

**Figure 2:**
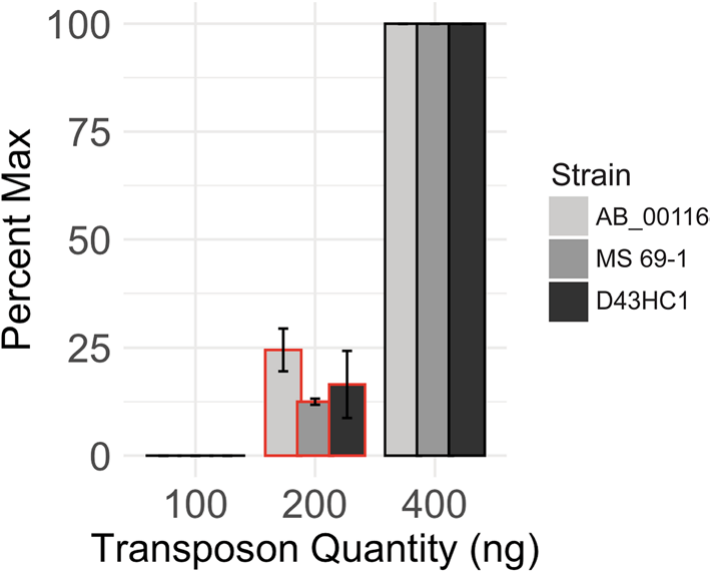
Higher quantity of transposon DNA during transposome assembly improves electroporation efficiency. We assembled the transposome by mixing 1U of Tn5 transposase with various amounts of the KAN2 transposon (100, 200, 400 ng). We electroporated our *E. coli* cohort and measured CFUs the following day. Bars indicate normalized CFUs as a function of transposon quantity during assembly. Error bars indicate variance among replicates (p = 7.95e–12). Bars with red highlights indicate the recommended transposon quantity in a standard TraDIS protocol (EZ-Tn5 Transposase manual).

We also examined how electroporation efficiency is affected by the quantity of transposon DNA during transposome assembly (Fig. 2). To analyze this, we assembled transposomes by mixing 1 U of Tn5 with 400, 200, or 100 ng of KAN2 transposons. We mixed 0.05 U of the assembled transposomes with electrocompetent cells, and assessed the electroporation efficiency by counting the resulting CFUs the next day. All conditions were performed in duplicate. The CFUs were the highest for those electroporated with the transposome with 400 ng KAN2 transposon, followed by 200 ng (18.0% CFU on average relative to 400 ng), and then 100 ng, which yielded virtually no colonies like the mock-electroporated negative control. We assessed the effect of transposon DNA quantity on electroporation efficiency by two-way ANOVA (p = 7.95e–12). The time constant (τ) was not affected by the transposon quantity.

### Cell density in electroporation reaction affects its efficiency

**Figure 3:**
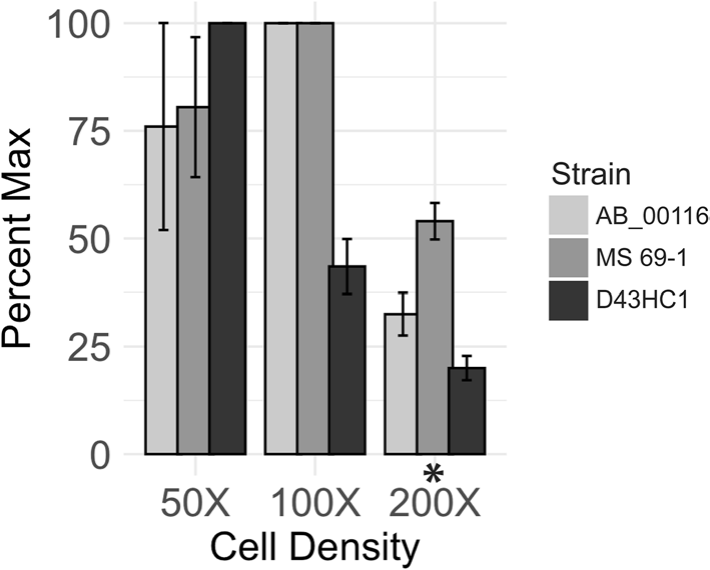
Cell density in electroporation reaction affects its efficiency**.** Prior to electroporation, we altered the cell density by resuspending cells in various volumes of water (50X, 100X, 200X concentrated from the starter culture volume). We electroporated our *E. coli* cohort with 0.05U of transposome and measured CFUs the following day. Bars indicate normalized CFUs as a function of cell density (p = 0.001). Error bars indicate variance among replicates. *p < 0.01.

We determined how electroporation efficiency is affected by the cell density in the reaction mixture (Fig. 3). We mixed 0.05 U of the transposomes with cell suspension of different densities (50X, 100X or 200X of the starter culture volume), and assessed the efficiency by counting the resulting CFUs the next day. All conditions were performed in duplicate. We found that overall, 100X concentrate of the starter culture volume had the highest electroporation efficiency, followed by 50X (21.4% less efficient on average) and 200X (56.8% less efficient on average; p = 0.0014). The density producing the highest electroporation efficiency was somewhat strain specific. The time constant (τ) was not affected by cell density.

### Increasing recovery time after electroporation does not significantly affect the number of unique insertion events

Electroporation of transposome into bacterial cells is immediately followed by a recovery period in rich growth medium such as SOC. This recovery period provides time for bacterial cells that successfully took up the transposome to express selection marker genes. However, the length of recovery time is not standardized in the TraDIS assay and varies among previous studies (from 1–2 h^4,5,7,8^).This is an important parameter to consider when creating the mutant strain library, as the number of unique mutants is often estimated from CFUs.

**Figure 4:**
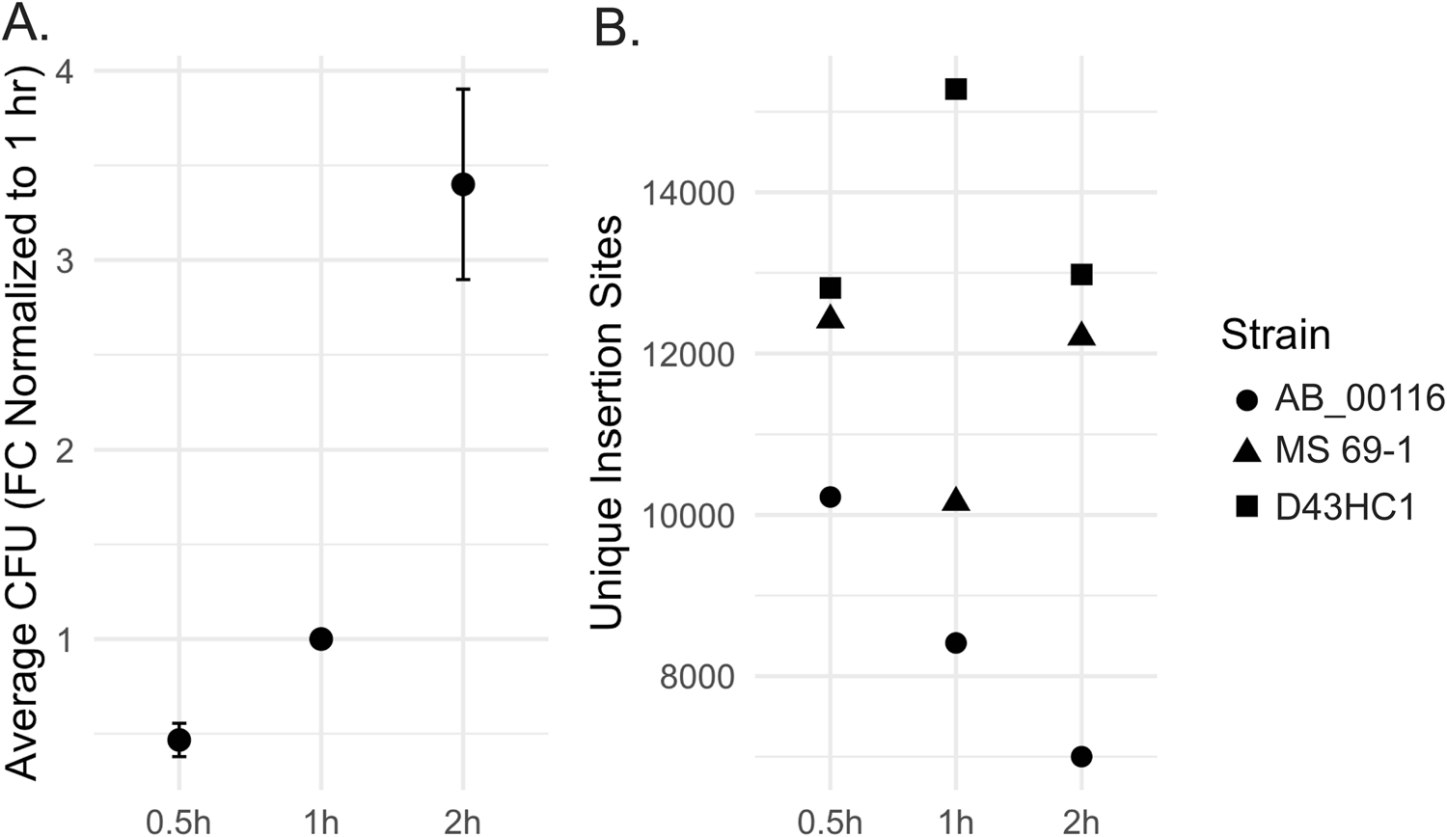
Altering recovery time after electroporation does not significantly affect the number of unique mutants: We electroporated our *Escherichia coli* cohort with 0.05U of Tn5 transposome and recovered for 0.5, 1 or 2 h before plating. We determined CFUs on the following day. (**A**) Average CFUs among the *E. coli* cohort at respective time points, which are normalized to CFUs at 1 h recovery time. *FC* fold change. (**B**) The number of unique insertion sites for each *E. coli* strain at respective time points (p = 0.4).

Therefore, we sought to examine the relationship between the number of CFUs and unique mutants, as a function of recovery time length. We hypothesized that longer recovery time would result in a lower proportion of unique mutants because longer recovery time would allow expansion of clones in culture. To test the hypothesis, we electroporated our *E. coli* cohort with the Tn5 transposome carrying the KAN2 gene, and let them recover for 0.5, 1, and 2 h before plating on kanamycin selection plates. After an overnight incubation, we harvested the same number of colonies for each time point per strain, and determined the number of unique mutants via sequencing. Not surprisingly, a longer recovery time yielded higher CFUs, doubling roughly every 0.5 h of recovery time (Fig. 4A). Contrary to our expectation, however, we did not observe a corresponding decrease in the number of unique insertion events (p = 0.4), and the number of unique mutants recovered was somewhat strain dependent (Fig. 4B). For two of the three test strains (MS 69-1 and D43HC1), we found that the number of unique mutants detected were comparable across time points; whereas, for strain AB_00116, we observed a reduced number of mutants as the recovery time lengthens.

### Selecting mutants in liquid broth may be a viable alternative to construct a complex mutant library

Constructing the TraDIS mutant library is a laborious task. To achieve the desired complexity of the library, it is necessary to prepare, plate, and scrape hundreds of agar plates to collect hundreds of thousands of colonies. A potential alternative to this method is to select the bacteria in a pooled fashion using liquid broth with a selection agent. Previously, Fels and colleagues developed a variant of the Tn-seq approach called Transposon Liquid Enrichment Sequencing (TnLE-seq) and demonstrated that a liquid broth can be successfully used to build a complex mutant strain library of *Desulfovibrio vulgaris* Hildenborough^9^. However, only a handful of studies have adopted the strategy^10,11^, despite its potential to save time, labor and cost.

We sought to examine whether the liquid broth approach could be adopted for constructing complex mutant libraries after electroporation of Tn5 mini-transposons. We electroporated our *E. coli* cohort with the Tn5 transposome carrying the KAN2 selection gene, and after 1 h of recovery, selected the resulting transformants either: (1) by plating them onto LB-kanamycin plates and collecting colonies after overnight incubation; or (2) by incubating them in a liquid broth with kanamycin and later collecting as the culture reached early-, mid-, or late log growth phase. All experiments were done in duplicate. We isolated DNA from each condition and determined the number of unique mutants via sequencing after subsampling reads to 5 million for each strain and condition (Table 1). For two of the three test strains (AB_00116 and MS 69-1), we found that the number of unique mutants detected were comparable between the two selection methods; whereas, for one of the test strains (D43HC1), we observed on average 15% reduction in the number of mutants when liquid broth was used for selection. For all strains, the growth phase of the liquid cultures at the time of harvest did not affect the number of unique mutants (0 = 0.44).

**Table 1:** The effect of selection medium on the number of unique insertion sites (UISs).

Strain	Method	Avg	Variance
AB_00116	Agar plate	100.0%	–
	Liquid (early)	104.2%	± 2.0%
	Liquid (mid)	104.5%	± 1.4%
	Liquid (late)	105.8%	± 2.0%
MS 69-1	Agar plate	100.0%	–
	Liquid (early)	107.1%	± 3.6%
	Liquid (mid)	106.7%	± 3.8%
	Liquid (late)	116.0%	± 5.7%
D43HC1	Agar plate	100.0%	–
	Liquid (early)	85.0%	± 0.2%
	Liquid (mid)	85.1%	± 0.1%
	Liquid (late)	85.0%	± 0.1%

Shown are relative % of UISs recovered using the agar plate method as reference. Liquid culture samples were harvested during early-, mid-, or late- exponential growth phases (2 replicates per condition).

### Nextera-TruSeq hybrid library design enables a simple PCR approach to enrich transposon-DNA junctions

**Figure 5:**
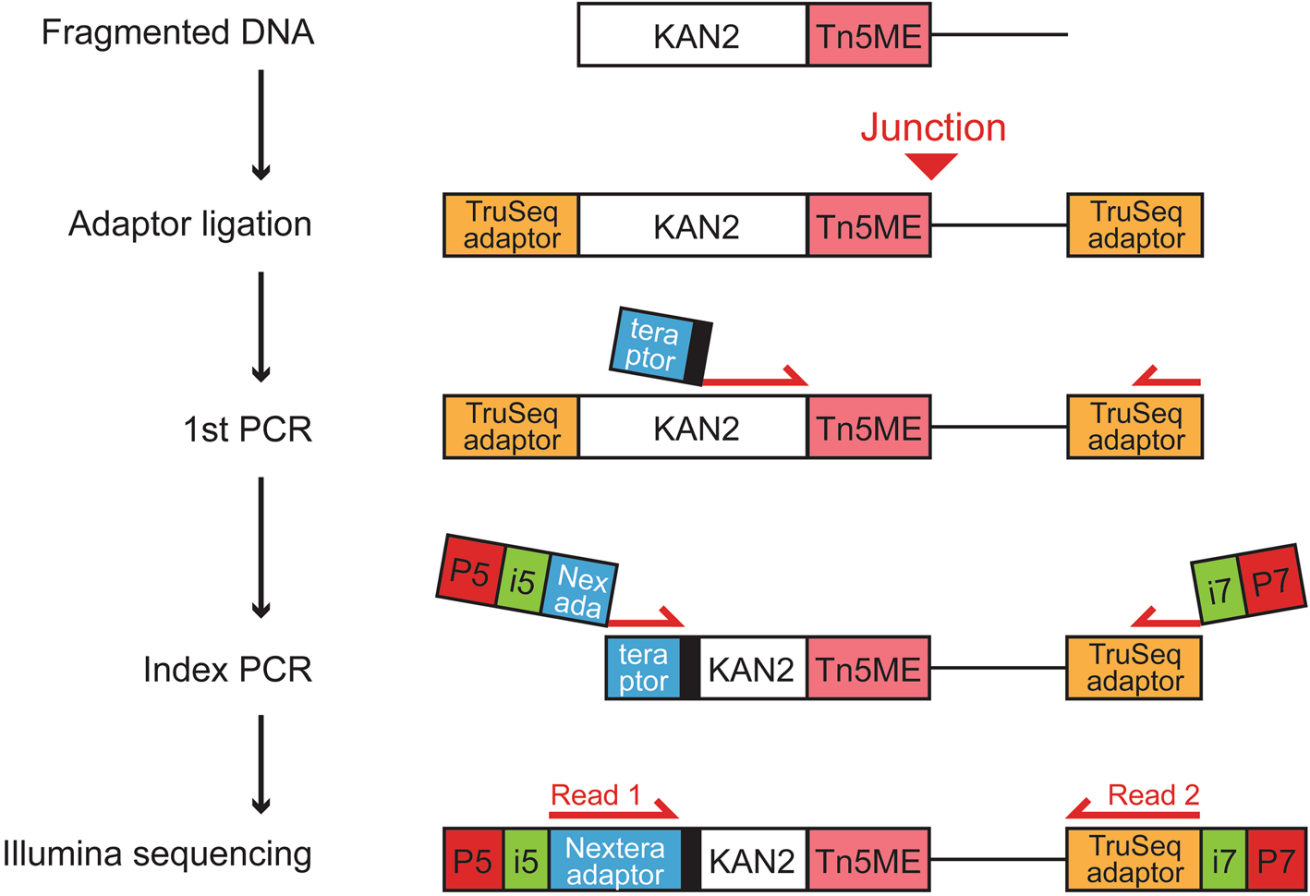
Schematics of simplified TraDIS library preparation. Following DNA fragmentation, a standard protocol was used to end repair, dA-tail, and ligate the TruSeq adaptors. Adaptor-ligated DNA samples were subjected to the 1st PCR using a transposon-specific primer with: (1) a partial Nextera i5 adapter (indicated by blue rectangle with ‘tera ptor’); (2) a ‘balancer’ (indicated by black rectangle); and (3) the anchor sequences that target the KAN2 transposon. The reverse primer was targeted to the TruSeq i7 adapter. Index PCR added necessary components for Illumina sequencing, such as i5/i7 indexes for sample multiplexing and P5/P7 flow cell adaptors. The resulting libraries were sequenced on the Illumina NovaSeq platform without custom sequencing primers or operation of the machine.

Our simplified workflow to prepare the TraDIS libraries is shown in Fig. 5. Following DNA fragmentation, we used a standard protocol to end repair, dA-tail, and ligate the TruSeq adaptors (see “Methods”). Adaptor-ligated DNA samples were then subjected to the first round of PCR using a transposon-specific primer (< 60 bp) that had three components: (1) a partial Nextera i5 adapter (34 bp); (2) a ‘balancer’ (3–6 bp) to increase nucleotide diversity during a sequencing run as previously done^4,5^; and (3) the anchor sequences that target the KAN2 transposon (20 bp). The reverse primer was targeted to the TruSeq i7 adapter. During indexing PCR, we added necessary components for Illumina sequencing such as i5/i7 indexes for sample multiplexing and P5/P7 flow cell adaptors (primer sequences are available in Supplemental Table 2). We sequenced the resulting libraries on the Illumina NovaSeq platform without custom sequencing primers or machine operation protocol, and then calculated the fraction of usable reads that contained transposon-DNA junctions (see “Methods” for filtering criteria and analysis workflow). Our simplified workflow generated a high fraction of usable reads (on average 79.3 ± 1.1% among 9 samples with at least 6 million reads each). Using the Nextera i5 adapter in the transposon-specific primer was crucial, as the fraction of usable reads plummeted to on average 0.34 ± 0.01% when we replaced the Nextera i5 adapter with the TruSeq i5 adapter. Poor performance of the TruSeq adaptor primer is likely due to non-specific amplification, as > 95% of non-usable reads were properly-paired and mappable to appropriate genomes (see “Discussion” for a potential cause).

## Discussion

Transposon insertion sequencing is a powerful high-throughput method of identifying essential bacterial genes in various growth conditions. Yet, there are several key technical hurdles that one has to overcome to successfully conduct the assay: an efficient construction of a complex mutant library, and a robust detection of the resulting transposon-DNA junctions using deep sequencing. In this study, we have identified a number of electroporation parameters that are readily adjustable that can enhance the number of transformants, and also described a simpler sequencing library workflow that is more cost-effective and flexible than previous approaches. We believe that these technical improvements can make this powerful technique more accessible for a wider audience.

We found that more dilute concentration of the Tn5 transposome was more effective in generating higher CFUs, at least among our cohort of three *E. coli* strains we tested, as the optimal concentrations ranged between 10 and 20 times lower than the concentration frequently used in prior TraDIS studies (1 U). Poorer performance at higher transposome concentrations is likely due to the carryover of the Tn5 storage buffer into electroporation reactions, whose salt content may interfere with its efficiency. In support of this, reactions with higher transposome quantity had parallel decrease in the time constant, which is often a good indicator of electroporation efficiency. On the other hand, overly diluted transposome solutions result in a low overall yield of viable mutants, although these dilutions do not necessarily compromise the electroporation process itself as indicated by time constant.

The ability to use more diluted transposomes has several advantages. First, since the commercially available Tn5 transposase is an expensive reagent of the TraDIS assay, this reduces the overall cost of each electroporation reaction by 10–20 fold. Second, because less amount of the enzyme is required for each round of electroporation, this allows researchers to optimize electroporation conditions directly using Tn5 transposomes, instead of using other molecules like plasmids as a proxy, whose optimized parameters may not be necessarily the same as those for Tn5 transposomes. Optimization of optical density (OD) and/or growth phase prior to electroporation is another well-documented factor influencing mutant yields (PMID: 35804085, PMCID: PMC3939052) that may vary between bacteria. As our experiments were specific to *E. coli*, we acknowledge that researchers applying these methods to other bacterial species might need to consider the OD/growth phase as a critical parameter. More experiments are required to see if our findings are generalizable to other microbial species.

We also observed that the transposon concentration during transposome assembly has a striking impact on CFU recovery. A standard protocol (EZ-Tn5 Transposase manual) suggests the pairing of 200 ng of a transposon with 0.5 µM of Tn5 transposase (53.3 kDa) during assembly. We found that doubling the quantity of transposon to 400 ng enhanced CFUs by roughly fourfold, while halving the quantity to 100 ng reduced CFUs virtually to none. Our findings clearly show that using an insufficient amount of transposon during transposome assembly is detrimental to obtaining viable mutants, and therefore, a proper calculation of the molar ratio between the Tn5 transposase and the transposon is crucial. In our most successful condition with 400 ng of the KAN2 transposon (1221 bp), the molar ratio during assembly was roughly 15:1 Tn5 transposase to the transposon. Because a complete transposome requires 2 molecules of Tn5 for every molecule of transposon, 400 ng of the KAN2 transposon would allow us to form at maximum ~ 15% of complete transposomes in the assembly mixture. Therefore, it is theoretically possible that adding more quantities of transposons can yield higher CFUs; however, we did not test this in the current study. Similar to the Tn5-to-transposon ratio during assembly, our analysis also found that the cell density during electroporation reactions is another factor that can impact CFUs. We found that concentrating a greater number of cells does not necessarily yield a higher number of viable mutants, and that there were strain-specific variations in the optimal densities.

When constructing the mutant libraries, CFUs are often used as a proxy for the number of unique mutants until sequencing is complete. While this is often a good proxy, existing TraDIS studies report a wide range of the CFU-to-unique mutant ratio (10–90%), and it is hard to draw conclusions about what parameters might be affecting this ratio. To address this, we investigated one potential factor influencing this relationship by varying the length of recovery time after electroporation. We reasoned that longer recovery time would result in higher CFUs but would also result in a lower proportion of unique mutants as longer incubation might facilitate expansion of clones. As expected, longer recovery time generated higher CFUs with an estimated doubling time of 0.5 h for our *E. coli* cohort. However, contrary to what we expected, our results showed that the number of unique mutants did not decline in proportion; therefore, a longer recovery time may increase the overall yield of unique mutants perhaps by allowing more time for transposomes to mutate the genome.

Constructing the TraDIS mutant library is a laborious and time-consuming task. Inspired by the success of TnLE-seq, a variant of Tn-seq method that uses liquid broth as a selection medium to construct a complex mutant library, we explored the possibility of adopting this approach to TraDIS. Based on the side-by-side comparison of the number of unique mutants obtained from agar plates or liquid broth, our results suggest that liquid broth may be a viable option for selecting mutants for the TraDIS assay. Because of the pilot nature of our study, we intentionally kept the complexity of our mutant libraries low (~ 10,000 unique mutants per condition). Future studies should address whether more complex libraries can be built using the liquid broth method.

Successful TraDIS assay also relies on efficient detection of transposon-DNA junctions via deep sequencing, and in this study, we introduced a simpler library design that does not involve the ligation of custom adaptors, custom sequencing primers during a sequencing run, or custom operation protocol of the sequencer such as the need for dark cycles. Despite its simplicity, our Nextera-TruSeq layout design generated a high proportion (~ 80%) of reads that correspond to transposon-DNA junctions. This is likely due to the ‘hybrid’ nature of the fragment layout because the same library preparation workflow failed if both ends of the fragments had TruSeq adaptors, as less than 0.5% of sequenced fragments corresponded to transposon-DNA junctions. The suboptimal performance of the TruSeq-TruSeq fragment layout can be attributed to the mechanics of the two-stage PCR process. Like previous approaches, the first round of PCR is designed to specifically amplify DNA fragments that contains transposon-DNA junctions; however, in the 2nd round of PCR, indexing primers are targeted to partial adaptor sequences that would also be present on all DNA fragments even if they do not contain junctions (Fig. 5). Consequently, depending on the relative proportion of target DNA and non-specific DNA fragments at the end of the first PCR, non-specific DNA can dominate the sequencing outputs. The Nextera-TruSeq hybrid layout minimizes this type of non-specific amplification during index PCR because only target junction fragments would have the partial Nextera adapter on their 5’ end.

Our library design is also simpler in terms of indexing, as it uses the standard TruSeq and Nextera indexing primers that are compatible with other Illumina sequencing assays. Therefore, adapting the assay to a different transposon simply involves replacing the primers in the first PCR to target transposon-specific sequences, which is more cost-effective than synthesizing long all-in-one primers. Although we did not directly demonstrate this versatility in the current study, our hybrid library design should be compatible using other DNA library preparation kits, such as Illumina DNA Prep kit that integrates Nextera adaptors during tagmentation. In this instance, one should set up the first round of PCR using a transposon-specific forward primer with the TruSeq i5 adaptor, and a reverse primer that targets the Nextera i7 adaptor to generate TruSeq-Nextera library fragments to minimize non-specific amplification.

TraDIS protocols still face several challenges that require improvement. Firstly, the efficiency and uniformity of transposon insertion across the genome needs enhancement to ensure comprehensive mutagenesis coverage. Even with strains that are amenable to genetic manipulation, the transformation efficiency using the transposome complex or plasmid vector, along with specific growth parameters and timing, can significantly limit mutant production and the accuracy of downstream analysis in TraDIS protocols. Secondly, data analysis pipelines must be optimized to identify and quantify insertional mutants accurately. Lastly, the development of more scalable library construction methods is necessary to facilitate the study of diverse organisms, enabling broader applications of TraDIS in functional genomics.

## Conclusion

In conclusion, we have identified several key areas for optimization that significantly reduce labor, time, and cost. These improvements should notably lower the entry barrier for newcomers to the technique.

### Supplementary Information

Supplementary Tables.

## Data Availability

Raw sequencing data and metadata are available at the NCBI Sequence Read Archive (SRA) under accession number PRJNA1055263.
